# Depression, anxiety, and suicidal ideation among Vietnamese secondary school students and proposed solutions: a cross-sectional study

**DOI:** 10.1186/1471-2458-13-1195

**Published:** 2013-12-17

**Authors:** Dat Tan Nguyen, Christine Dedding, Tam Thi Pham, Pamela Wright, Joske Bunders

**Affiliations:** 1 Faculty of Public Health, Can Tho University of Medicine and Pharmacy, Can Tho, Vietnam; 2 VU University, Amsterdam, The Netherlands; 3 The Medical Committee Netherlands-Vietnam (MCNV), Hanoi, Vietnam

**Keywords:** Mental health, Depression, Anxiety, Suicide, Academic pressure, Adolescents, Students, Vietnam, Secondary schools

## Abstract

**Background:**

There is a rapidly growing public awareness of mental health problems among Vietnamese secondary school students. This study aims to determine the prevalence of anxiety, depression, and suicidal ideation, to identify related risk factors, and to explore students’ own proposals for improving their mental health.

**Methods:**

A cross-sectional study was conducted among 1161 secondary students in Can Tho City, Vietnam during September through December, 2011. A structured questionnaire was used to assess anxiety, depression, suicidal ideation and proposed solutions. Depression was measured using the Center for Epidemiology Studies Depression Scale.

**Results:**

The prevalence estimates of symptoms reaching a threshold comparable to a diagnosis of anxiety and depression were 22.8% and 41.1%, respectively. Suicide had been seriously considered by 26.3% of the students, while 12.9% had made a suicide plan and 3.8% had attempted suicide. Major risk factors related to anxiety and depression were physical or emotional abuse by the family, and high educational stress. As proposed solutions, nearly 80% of students suggested that the academic workload should be reduced and that confidential counselors should be appointed at schools. About half the students stated that the attitudes of their parents and teachers needed to change. A significant majority said that they would visit a website that provided mental health support for students.

**Conclusions:**

Anxiety, depression, and suicidal ideation are common among Vietnamese secondary school students. There are strong associations with physical and emotional abuse in the family and high educational stress. Academic curricula and attitudes of parents and teachers need to be changed from a punitive to a more supportive approach to reduce the risk of poor mental health. An internet-based mental health intervention could be a feasible and effective first step to improve students’ mental health.

## Background

Mental health disorders are among the most important public health issues globally. Estimates of the global burden of disease place mental illness in the top three conditions in terms of years lost due to disability [1]. The mental health of adolescents and young people is a crucial issue because of the general burden of mental illness and because it has the potential to affect their adult lives, and the lives of the next generation [2,3]. The problems that adolescents and young people encounter interfere with the way they think, feel, and act. Such problems cause distress and limit their academic achievements and ability to be economically productive. They can also lead to family conflicts, substance abuse, violence, eating disorders and sometimes suicide. Mental health problems are also expensive for families, communities, and healthcare and social systems [3].

There is a rapidly growing public awareness of mental health problems such as stress, anxiety, depression and suicide among Vietnamese students [4-6]. In the *Bulletin of the World Health Organization* 2006, Harpham and Tran reported that a fifth of young Vietnamese people experience mental health problems [7]. A cross-sectional study of 972 secondary school students, 13 to 16 years old, in the north of Vietnam showed that a high proportion had poor mental health, with 17.6% having felt sad and hopeless every day for two weeks during the past 12 months [8]. In addition, the number of students that had considered suicide was high, with 6.6% of students having seriously considered suicide during the past 12 months, 1.2% having made a suicide plan, and 0.4% having attempted suicide [9]. Three recent studies of urban youth - 2591 adolescents in Hanoi (2006) [10], approximately 1000 adolescents in Hanoi (2007), [8], and 410 university students in Ho Chi Minh City (2009) [11], revealed ranges of suicide ideation from 9.2-10.6%. Another study of 1226 secondary school students conducted in Ho Chi Minh City indicated that the percentages of students who had seriously considered suicide, planned to commit suicide or actually attempted suicide during the past 12 months was 6.3%, 4.6% and 5.8% respectively [12]. In addition, the prevalence of depression, anxiety and psychological distress was 26.3%, 16.2% and 36%, respectively. These studies demonstrate the high prevalence of depression, anxiety and educational stress among adolescents, as well as the strong association between educational stress and poor mental health. However, risk factors for depression and anxiety, and students’ perspectives on how to reduce depression or anxiety, have not yet been investigated.

Although depression and anxiety among students are prevalent disorders, very few studies have examined the prevalence of the conditions, the related risk factors, and youth’s proposed solutions. Therefore, the aim of this study was to determine the prevalence of anxiety, depression and suicide ideation in Vietnamese secondary school students, to identify risk factors, and to explore possible solutions to improve their mental health.

## Methods

### Study design and population

A cross-sectional study design was applied. All data were collected during the first academic semester (September to December, 2011). The sample was purposively selected from three secondary schools in urban and suburban areas in Can Tho City, Vietnam comprising specialized and general secondary schools. In each school, three classes, one from each grade from 10 through 12, were chosen randomly. In total, 1260 students were invited to participate in the study by sending an anonymous self-reporting questionnaire. No exclusion criteria, such as demographic and/or socioeconomic characteristics, were applied. However, of the 1260 students, 99 (7.85%) were excluded from our analysis due to insufficiently complete responses.

### Data collection

Self-completed questionnaires were distributed to all participants, who were requested to complete the questionnaire anonymously after class or at home to minimize potential sharing. The questionnaire consisted of five components: (1) demographic information including student’s family characteristics (parent educational and occupational profiles, parental marital status, family financial situation, and style of upbringing (how parents convey social norms and a sense of love and acceptance); (2) mental health scales (the Center for Epidemiology Studies Depression Scale – CES-D, and an anxiety scale); (3) the Educational Stress Scale for Adolescents (ESSA); (4) questions on suicide; and (5) questions on solutions.

The Center for Epidemiology Studies Depression Scale consists of 20 calibrated items, with high internal consistency with Cronbach’s alpha coefficients ranging from 0.85 to 0.90 among general population samples [13]. Items are scored either 0–3 or 3–0, with a range of total scores from 0–60; a higher score indicating a higher level of depression. This scale has been validated in Vietnam using confirmatory factor analysis [14]. The Center for Epidemiology Studies Depression Scale has a four-factor structure including depressed affect (7 items), “somatic and retarded activity” (7 items), positive affect (4 items) and interpersonal activity (2 items) [13]. The standard cut-off score of 16 was used to detect possible cases of depression [15]. For comparison with other studies, two other cut-off points were also used: scores over 21 (for depressive symptoms) and over 25 (for depression) [16,17].

The anxiety scale [14] consists of 13 items using a 3-point scale (never, sometimes, often). The scale showed a high level of internal consistency (Cronbach’s alpha ranged from 0.76 to 0.81) and has also been validated for use among Vietnamese students. The scale was assessed as providing a highly reliable measure of anxiety when used in a community survey of Vietnamese adolescents, showing a high internal consistency (Cronbach’s alpha = 0.82) [14]. A receiver operating characteristic (ROC) analysis was done in a study by Thai [12] to choose a cut-off point for further analysis using the Kessler 10 – Psychological Distress Scale as a reference standard. The chosen threshold was 26 with specificity of 92.2% and sensitivity of 31.3% (AUC = 0.72) [12] which was similar to psychometric properties of Kessler 10 [18,19].

The Educational Stress Scale for Adolescents (ESSA) consists of 16 items and uses a 5-point Likert scale ranging from 1 (strongly disagree) to 5 (strongly agree). The scale was used to measure educational stress, with higher scores indicating greater stress. It covers five dimensions including pressure from study (4 items), worry about grades (3 items), despondency (3 items), self-expectation (3 items) and workload (3 items) [12]. The ESSA has been validated to measure the educational stress of adolescents in Vietnam with a high level of internal consistency with a Cronbach’s alpha of 0.83 [20]. The internal consistency in the present study has a Cronbach’s alpha of 0.86. The cut-off points suggested by for Vietnam by Thai comprise <50 (low stress), 51–58 (medium stress) and >58 (high stress) [12].

In order to address the issue of suicide, additional questions about ever having seriously considered suicide and the making of a suicide plan, employed of a scale ranging from never, sometimes and often. A yes/no question was also used to identify students who had attempted suicide.

Finally, closed-open questions about possible solutions were used to explore students’ perspectives on improving mental health.

### Statistical analysis

Data are presented as means ± standard deviation (SD) and analyzed descriptively to determine the demographic and basic characteristics of the study population. The Chi squared test (χ2) was used to assess the significance of differences in the distribution of selected socio-demographic characteristics, risk factors, and outcome variables among the participants. In addition to the descriptive analyses, logistic regression analysis was performed to identify associations between depression or anxiety and family characteristics, educational stress, and academic achievement. Univariate independent predictors of anxiety and depression with p < 0.10 were entered in a multivariate logistic regression model, applying the Backward Wald method to study their influence on the presence of anxiety and depression. Predictors of anxiety included abuse (physical or emotional abuse by adults in the household or by teachers or other staff members at school), low academic performance from the previous semester, and educational stress. Predictors of depression included growing up and living with non-biological parents, living with a mentally ill person, being physically or emotionally abused by adults in the household, low academic performance from the previous semester, often having a serious quarrel with teachers in school, and being physically or emotionally abused by teachers or other staff at school.

All tests were 2-tailed and a p-value of <0.05 was considered statistically significant. The 95% confidence intervals of Odds Ratios (OR) were also calculated. To address the validity of Chi-square results, all the variables in all models must also have an expected cell frequency above 10 before entering them into the logistic regression model. Each model was also checked for goodness-of-fit by checking the significant value of the Hosmer-Lemeshow which must be higher than 0.05. All analyses were performed with SPSS, Version 16.0.

### Ethics

Ethical approval for the study was obtained from the Scientific and Technical Committee of the Can Tho University of Medicine and Pharmacy, which has authority to approve both technical content and ethical aspects of studies done by its staff and students.

## Results

The research population consisted of 1159 secondary school students including 424 (36.5%) boys and 737 (63.5%) girls, ranging in age from 15 to19 years old (overall mean age 16.1 years). The difference in the number of girls and boys reflects the actual population in the classes selected. The number of students for each grade from 10 to 12 was equal among the three grades, with a response rate of about 33% (grade 10: 33.5%, grade 11: 33.9%, and grade 12: 32.6%). The majority of students (95.3%) were ethnically Kinh (ethnic majority in Vietnam); other ethnic groups included Chinese and Khmer.

### Mental health

#### Anxiety

Scores on the 13-item anxiety scale based on current feeling range from 13–39, with a mean score (±SD) of 22.6 (± 4.19). Twenty three percent of students reported anxiety symptoms at a clinically significant level. Female students had three times the odds of having anxiety symptoms as compared to male students (29% vs. 12.1%) (OR = 2.94; 95% CI OR = 2.13-4.15, p < 0.001).

#### Depression

Scores on the 20-item CES-D Scale during the past week ranged from 0–55, with a mean score (±SD) of 15.7 (±10.5). The prevalence of being in a category at risk for clinical depression (a CES-D score of ≥ 16) was approximately 41.1%. In comparison with other studies, two other cut-off points were also used: scores over 21 (for an elevated level of depressive symptoms) and over 25 (for a level of depressive symptoms comparable with major depressive disorder) [16,17]. Accordingly, approximately one fourth (25.9%) of the students were classified as having an elevated level of depressive symptoms, while 18.7% demonstrated a level of depressive symptoms consistent with major depressive disorder. The results also showed that female students had a significantly higher level of depressive symptoms comparable with major depressive disorder and an elevated level of depressive symptoms (Table 1).

**Table 1:** Prevalence of depression among secondary school students

	**Total N (%)**	**Females N (%)**	**Males N (%)**	**OR (95% CI)**	**p**
*Risk of depression (CES-D score of ≥ 16)*					
Yes	476 (41.1)	324 (44.1)	152 (35.9)	**1.41 (1.10-1.80)**	**<0.007**
No	682 (58.9)	411 (55.9)	271 (64.1)	-	-
*Depressive symptoms (CES-D score of > 21)*					
Yes	300 (25.9)	209 (28.4)	91 (21.5)	**1.45 (1.09-1.92)**	**0.010**
No	858 (74.1)	526 (71.6)	332 (78.5)	-	-
*Depression (CES-D score of >25)*					
Yes	216 (18.7)	151 (20.5)	65 (15.4)	**1.42 (1.04-1.96)**	**0.029**
No	942 (81.3)	584 (79.5)	358 (84.6)	-	-

The Odds ratios with a CI not including 1.00 (before round-off) are given in bold.

### Factors associated with anxiety and depression

#### Factors associated with anxiety

Multivariate logistic regression analysis was performed to study the relationship between anxiety and several familial and educational factors. Anxiety was shown to be independently associated with: experiencing physical and emotional abuse within the family; physical and emotional abuse from teachers or other staff members at school; and high educational stress (Table 2).

**Table 2:** Risk factors for anxiety

**Characteristics**	**Anxiety**		**Univariate logistic regression**		**Multivariate logistic regression**	
	**Yes n (%)**	**No n (%)**	**OR (95% CI)**	**p**	**OR (95% CI)**	**p**
Being abused by parents, or other adults in the household* (n = 1152)						
No	223 (21.4)	819 (78.6)	-	-	-	-
Yes	39 (35.5)	71 (64.5)	**2.02 (1.33-3.06)**	**0.001**	**1.58 (1.02-2.45)**	**0.043**
Being abused by teachers or other staff members at school* (n = 1150)						
No	175 (20.4)	681 (79.6)	-	-	**-**	**-**
Yes	88 (29.6)	209 (70.4)	**1.64 (1.22-2.21)**	**0.001**	**1.46 (1.06-2.00)**	**0.019**
*Educational stress (n = 1148)*						
Low stress	44 (13.3)	288 (86.7)	-	-	**-**	**-**
Medium stress	66 (16.8)	328 (83.2)	1.32 (0.87-1.99)	0.191	1.31 (0.87-2.00)	0.193
High stress	153 (36.3)	269 (63.7)	**3.72 (2.56-5.41)**	**<0.001**	**3.49 (2.39-5.09)**	**<0.001**
**Total (1155)**	**263 (22.8)**	**892 (77.2)**				

*Participants were physically or emotionally abused by parents or other adults in the household, or by teachers or other staffs at school in the past 12 months*.* The Odds ratios with a CI not including 1.00 (before round-off) are given in bold*.*

#### Risk factors associated with risk of depression

Logistic regression was also used to explore the relation between depression and risk factors. The standard cut-off score of 16 in CES-D was used to identify possible cases of depression. Based on univariate logistic regression analysis, 12 variables significantly increased the risk of depression, while one variable (having a personal tutor) significantly decreased the risk (Table 3). Students were likely to show higher depressive symptoms when they were not living with both biological parents, living with alcohol or drug abusers, living with a mentally ill person, being physically or emotionally abused, and often having serious quarrels with teachers or other school staff members. Those with poor academic performance and high educational stress also showed more depressive symptoms. In contrast, having personal tutors reduced the likelihood of having depressive symptoms by 28%.

**Table 3:** Risk factors for possible case of depression

**Characteristics**	**Depression**		**Univariate logistic regression**		**Multivariate logistic regression**	
	**Yes n (%)**	**No n (%)**	***OR (95% CI)***	***p***	***OR (95% CI)***	***p***
*Growing up and living with both natural parents (n = 1158)*						
Yes	430 (40.0)	645 (60.0)	-	-	-	-
No	46 (55.4)	37 (44.6)	**1.86 (1.19-2.92)**	**0.007**	**1.88 (1.13-3.13)**	**0.015**
*Living with a substance abuser (n = 1154)*						
No	373 (38.5)	597 (61.5)	-	-	-	-
Yes	101 (54.9)	83 (45.1)	**1.94 (1.42-2.68)**	**<0.001**	**1.63 (1.13-2.34)**	**0.008**
*Living with a depressed or mentally ill person (n = 1154)*						
No	442 (40.2)	657 (59.8)	-	-	-	-
Yes	32 (58.2)	23 (41.8)	**2.07 (1.19-3.58)**	**0.008**	**1.93 (1.03-3.61)**	**0.041**
*Being abused by parents or other adults in the household (n = 1155)*						
No	405 (38.8)	640 (60.2)	-	-	-	-
Yes	70 (63.6)	40 (36.4)	**2.76 (1.84-4.16)**	**<0.001**	**1.80 (1.23-2.88)**	**0.014**
*Academic performance from the last semester (n = 1138)*						
Excellent/good	77 (33.6)	152 (66.4)	-	-	-	-
Fairly Good/average	350 (40.8)	508 (59.2)	**1.36 (1.00-1.85)**	**0.046**	1.25 (0.89-1.76)	0.192
Below average/ Very poor	39 (76.5)	12 (23.5)	**6.42 (3.18-12.95)**	**<0.001**	**3.95 (1.83-8.56)**	**0.001**
*Academic stress (n = 1151)*						
Low stress	86 (25.7)	248 (74.3)	**-**	**-**	**-**	**-**
Medium stress	129 (32.9)	263 (67.1)	**1.41 (1.02-1.95)**	**0.036**	**1.59 (1.12-2.25)**	**0.009**
High stress	259 (60.9)	166 (39.1)	**4.50 (3.29-6.15)**	**<0.001**	**5.02 (3.57-7.07)**	**<0.001**
*Having personal tutors (n = 1151)*						
No	103 (47.7)	113 (52.3)	**-**	**-**	**-**	**-**
Yes	370 (39.6)	565 (60.4)	**0.72 (0.53-0.97)**	**0.043**	**0.66 (0.47-0.92)**	**0.014**
*Serious quarrel with teachers or other staff at school in the past 12 months (n = 1155)*						
Never	367 (38.1)	597 (61.9)	-	-	-	-
Sometimes/often	107 (56.0)	84 (44.0)	**2.07 (1.51-2.84)**	**<0.001**	**1.92 (1.35-2.75)**	**<0.001**
*It is acceptable for students to have premarital sex (n = 1151)*						
No	394 (38.8)	622 (61.2)	-	-	-	-
Yes	80 (59.3)	55 (40.7)	**2.30 (1.59-3.31)**	**<0.001**	**2.12 (1.40-3.20)**	**<0.001**
**Total**	**476 (41.1)**	**682 (58.9)**				

*Participants were physically or emotionally abused by parents or other adults in the household, or by teachers or other staffs at school in the past 12 months. The Odds ratios with a CI not including 1.00 (before round-off) are given in bold.

In multivariable logistic regression analysis, not accounting for effect modification, nine variables still remained associated with possible depression. School marks that were considerably below average and high educational stress were the two strongest factors increasing the risk of depressive symptoms (odds ratios were 3.95 and 5.02, respectively). A personal tutor remained a protective factor for depression (reduced the odds by 34%) (Table 3).

### Associated with poor mental health

#### Suicidality

About one fifth of the students had seriously considered attempting suicide (24.7% indicated sometimes and 1.6% indicated often) and about one eighth (12% sometimes and 0.9% often) had made a suicide plan at some time. Forty-four (3.8%) of the 1144 students had attempted suicide at least once.

#### Association between suicidal ideation and poor mental health

Students with anxiety and depression were at a higher risk of suicidal ideation. Depression was the strongest predictor of suicide planning according to both univariate and multivariate logistic analysis, and anxiety and depression were the main predictors of suicide attempts (Table 4).

**Table 4:** Association between suicidal ideation and poor metal health

	**Suicidal behaviors**		**Univariate logistic regression**		**Multivariate logistic regression**	
	***Sometimes/often n (%)***	***Never n (%)***	***OR (95% CI)***	***P***	***OR (95% CI)***	***p***
***Seriously considered suicide***	***304 (26.3)***	***850 (73.7)***				
*Anxiety (n = 1152)*						
Low anxiety	186 (20.9)	703 (79.1)	-	-	-	-
High anxiety	118 (44.9)	145 (51.1)	**3.08 (2.30-4.12)**	**<0.001**	**1.84 (1.33-2.57)**	**<0.001**
*Depression (n = 1154)*						
Low depressive risk	112 (16.5)	568 (83.5)	-	-	-	-
Possible case of depression	192 (40.5)	282 (59.5)	**3.45 (2.63-4.54)**	**<0.001**	**2.69 (1.98-3.64)**	**<0.001**
*Sex (n = 1157)*						
Male	73 (17.3)	349 (82,7)	**-**	**-**		
Female	231 (42.4)	504 (68,6)	**2.19 (1.63-2.95)**	**<0.001**	**2.31 (1.66-3.24)**	**<0.001**
*Drinking alcohol (n = 1153)*						
None drinker	200 (22.6)	685 (77.4)	**-**	**-**	**-**	**-**
Drinker	104 (38.8)	164 (61.2)	**2.17 (1.62-2.91)**	**<0.001**	**2.48 (1.77-3.48)**	**<0.001**
*Being abused by parents or other adults in the household (n = 1154)*						
No	247 (23.7)	797 (76.3)	**-**	**-**	**-**	**-**
Yes	56 (50.9)	54 (49.1)	**3.35 (2.24-4.99)**	**<0.001**	**2.86 (1.83-4.47)**	**<0.001**
*Schools (n = 1157)*						
TDN	84 (21.6)	305 (78.4)	**-**	**-**	**-**	**-**
CVL	114 (27.2)	305 (72.8)	1.36 (0.98-1.88)	0.064	**1.55 (1.08-2.24)**	**0.019**
LTT	106 (30.4)	234 (69.6)	**1.58 (1.14-2.21)**	**0.007**	**2.11 (1.45-3.10)**	**<0.001**
*Having tutor after school (n = 1150)*						
No	45 (21.0)	160 (79.0)	**-**	**-**	**-**	**-**
Yes	257 (27.5)	679 (72.5)	1.42 (0.99-2.04)	0.054	**1.52 (1.02-2.26)**	**0.040**
*Serious quarrel with teachers or other staff at school in the past 12 months (n-1154)*						
Never	236 (24.5)	726 (75.5)	**-**	**-**	**-**	**-**
Sometimes and often	68 (35.4)	124 (64.6)	**1.69 (1.21-2.35)**	**0.002**	1.42 (0.97-2.97)	0.068
***Planning suicide***	***144 (12.9)***	***972 (87.1)***				
*Depression (n = 1116)*						
Low depressive risk	36 (5.5)	621 (94.5)	-	-	-	-
Possible case of depression	108 (23.5)	351 (76.5)	**5.31 (3.56-7.91)**	**<0.001**	**4.44 (2.93-6.76)**	**<0.001**
*Sex (n = 118)*						
Male	40 (9.8)	369 (90.2)	-	-	-	-
Female	104 (14.7)	605 (85.3)	**1.59 (1.08-2.34)**	**0.020**	**1.73 (1.13-2.62)**	**0.012**
*Grade (n = 118))*						
10	44 (11.8)	330 (88.2)	-	-	-	-
11	62 (16.5)	314 (83.5)	1.48 (0.98-2.45)	0.064	1.44 (0.92-2.29)	0.112
12	38 (10.3)	330 (89.7)	0.86 (0.55-1.37)	0.532	0.77 (0.46-1.27)	0.300
*Drinking alcohol (n = 1116)*						
None drinker	97 (11.3)	758 (88.7)	-	-	-	-
Drinker	47 (18.0)	214 (82.0)	**1.72 (1.17-2.51)**	**0.005**	**1.56 (1.02-2.38)**	**0.038**
*Serious quarrel with teachers or other staff at school in the past 12 months (n = 1115)*						
Never	103 (11.1)	828 (88.9)	-	-	-	-
Sometimes and often	41 (22.3)	143 (77.7)	**2.30 (1.54-3.45)**	**<0.001**	**1.88 (1.20-2.95)**	**0.006**
*Being abused by parents or other adults in the household (n = 1115)*						
No	106 (10.5)	903 (89.5)	-	-	-	-
Yes	37 (34.9)	69 (65.1)	**4.57 (2.92-7.14)**	**<0.001**	**3.33 (2.04-5.44)**	**<0.001**
*Having tutor after school (n = 1111)*						
No	123 (13.7)	778 (86.3)	-	-	-	-
Yes	20 (9.5)	190 (90.5)	1.50 (0.91-2.47)	0.108	**1.77 (1.04-3.01)**	**0.036**
***Attempted suicide***	***44 (3.9)***	***1095 (96.1)***				
*Anxiety (n = 1139)*						
Low anxiety	21 (2.4)	859 (97.6)	-	-	-	-
High anxiety	23 (8.9)	236 (91.1)	**3.97 (2.17-7.33)**	**<0.001**	**2.68 (1.42-5.08)**	**0.002**
*Depression (n = 1141)*						
Low depressive risk	11 (1.6)	661 (98.4)	-	-	-	-
Possible case of depression	33 (7.0)	436 (93.0)	**4.55 (2.27-9.10)**	**<0.001**	**3.39 (1.65-6.98)**	**0.001**
*Drinker*						
*Drinking alcohol (n = 1141)*						
None drinker	223 (2.6)	854 (97.4)	-	-	-	-
Drinker	21 (8.0)	243 (92.0)	**3.21 (1.75-5.88)**	**<0.001**	**2.95 (1.58-5.49)**	**0.001**

*Participants were physically or emotionally abused by parents or other adults in the household, or by teachers or other staffs at school in the past 12 months. The Odds ratios with a CI not including 1.00 (before round-off) are given in bold.

### Student’s proposed solutions

Students proposed a number of measures to improve mental health. First, nearly four fifths of the students thought that the demands of the academic curricula should be reduced (79.8%), and that schools should have a confidential counselor to help them (78.8%). About half thought their parents’ (47.6%) and teachers’ (43.9%) attitudes and behaviors toward them needed to change and that teacher should take a supportive rather than punitive approach. Approximately 64% of respondents strongly agreed that students would share private problems and seek help on a suitable website. Most (90%) of the respondents said they would visit such a website if it existed. Finally, some students wanted parents and teachers to be supported in gaining counseling skills so that they could better sympathize with and help students at risk for mental health problems.

## Discussion

This study demonstrates that, according to their own responses, nearly one fourth (22.8%) of secondary school students in Can Tho are at risk of anxiety, and two fifths (41.1%) are at risk for depression. Female students reported higher levels of anxiety and depression. Key risk factors were physical and emotional abuse within the household, poor school performance and high educational stress. Anxiety and depression were found to be strong predictors of suicidal ideation. As many as 26.3% of students had seriously considered suicide, 12.9% had made a suicide plan and 3.8% had attempted suicide. A majority of students thought that reducing the demands of the academic curriculum, appointing confidential counselors and sharing their concerns on an appropriate website would help to improve their mental health.

The prevalence of anxiety was higher than in previous studies of secondary school students in Ho Chi Minh City (16.2%) [12]. The prevalence of depressive symptoms was 25.9% based on CES-D scores with the cut-off point >21, and 18.7% using the cut-off point >25. The prevalence of depressive symptoms in this study was quite similar to Thai’s findings (2010) (26.3%) but much higher than that of a study of adolescents (12–17 years) in California, USA, in which approximately 10% of the adolescents reported more than 10 depression symptoms [21]. However, another US study reported a higher level of depressive symptoms among young people (mean age 19.7) with 38.5% and 10.4% scoring at or above 16 and 28 CES-D total scale cutoffs respectively [22]. Regional and national differences in the mental health scores of children and adolescents may be explained by several factors including individual, familial, and environmental/cultural. Environmental stressors, such as poverty, traumatic events and illness, have been consistently linked to poor mental health among youth around the globe [23]. The socio-economic development status in Can Tho City, the location of this study, is much lower than in Ho Chi Minh City or the USA, which may explain the higher levels of anxiety and depressive symptoms.

The results of this study revealed one potentially important difference between the prevalence of mental health problems among Vietnamese students in Can Tho City (South of Vietnam) and in Da Nang and Khanh Hoa provinces (Central Vietnam). In this study, female students had a significantly higher risk of anxiety and depression while the study of Da Nang and Khanh Hoa provinces, undertaken in 2006, found that gender was not significantly associated with overall rates of anxiety and depression among Vietnamese youth [24]. However, it is important to note that the previous study only evaluated gender difference in relation to adolescents’ total Strengths & Difficulties Questionnaire (SDQ) scores. The present study showed a trend consistent with prevalence estimates of mental health problems among adolescents in Vietnam and in Western countries.

The prevalence of suicidal thoughts (26.3%) and suicide plans (12.9%) in this study population was higher than in the Ho Chi Minh City (6.3% suicidal thoughts and 4.6% suicidal plans) and Hanoi studies (10.6% suicidal thoughts) [8], but similar to levels found among Malaysian secondary students (25.3% with suicidal thoughts and 10.5% with suicide plans) [25]. The prevalence of suicide attempts however (3.8%), was lower than in Ho Chi Minh City (5.8%) [12] and Hanoi (9.2%) [10]. Suicide featured more prominently among these students than among first year university students at Can Tho University of Medicine and Pharmacy [9], although both study populations were living in the same region. Lifestyle differences between Can Tho, Ho Chi Minh City and Hanoi may be responsible for differences in mental health status (e.g. economic status and other factors as mentioned above). The higher rate of suicidal ideation among secondary school students in this study compared to medical university students may reflect the higher education and income of university students’ parents. Another interpretation could be that the medical students are farther along in their studies and may be more confident of their outcomes, whereas the secondary school students may still be comparatively insecure.

Previous research [12] found that high academic pressure and recent poor academic performance were associated with higher levels of depressive symptoms. In this study, high educational stress was also a main predictor of anxiety and depression among students, consistent with findings from a national study [6,12] and from international studies [26-28].

Our findings also suggest, perhaps not surprisingly, a relationship between abuse and mental health problems. Our results also confirmed that frequent physical or emotional abuse from adults (parents or other adults in the family, teachers or other staff members at school) was an independent predictor of anxiety. This finding was similar to other recent studies in Ho Chi Minh City [12] in Hanoi and in rural Hai Duong [29]. This is also consistent with findings from other studies in Western countries that demonstrate that maltreatment in childhood predicted difficulties in psychological adjustment in adolescence [30,31].

Nearly 80% of students thought that mental health problems could be improved by lightening the academic curriculum and by the appointment of a confidential counselor for students at school. These are new findings for which we did not find comparable results. Reducing the academic curriculum is not a quick and easy matter because it is a process involving thousands of schools around the country, not only the schools in the study. The appointment of confidential counselors for students is also difficult to implement because schools in Vietnam lack staff and financial support for such providers. However, most of the students reported that they would share their private problems and seek help from a website if it was available. Developing such a website should be feasible in order to provide an internet-based psycho-educational intervention for students in Vietnam.

### Limitations of the study

Several limitations of the study need to be considered. First, the CES-D scale, like other screening instruments, cannot be viewed as a diagnostic tool but only as a screening instrument to identify members of groups at risk of depression because it has relatively low specificity - 77% in primary care patients using the cut-off point of 16 [15,32]. Second, the temporal sequence of covariates and depression, for example, cannot be ascertained because it was a cross-sectional study.

Third, non-response bias should also be taken into account. In general, people who participate in health surveys are healthier than those who do not [33]. Thus, the levels of depressive symptoms, anxiety, and suicide ideation are probably underestimated. However, the difference between respondents and non-respondents were similar in all subgroups in the study sample so the comparisons between populations are valid, even if the absolute prevalence rates may be underestimated [33]. The low non-respondent bias should not affect the association between depressive symptoms or anxiety and risk factors within the study sample.

Fourth, the study population may not be representative of all youth of secondary school age throughout the country, given that some youth do not attend secondary school. However, compared with the national average, rates of depression and anxiety were quite similar to national studies [12]. Another limitation is that the cut-off scores were not validated among youth in Vietnam which means they should be used with caution. Finally, the low sensitivity of the anxiety measure (31%) may have resulted in an underestimate of the burden of anxiety.

## Conclusions

The rates of anxiety and depression found confirm the findings of other studies that the prevalence is high among Vietnamese secondary school students. Research shows that anxiety and depression have significant effects on students’ quality of life, and are major risk factors for suicide. Emotional abuse within the family and high educational stress were the main predictors of anxiety and depressions. The most feasible strategy in reducing mental health problems and promoting mental health among secondary school students in Vietnam today would be the development of a website to provide psycho-educational interventions designed to meet the needs of Vietnamese youth. In addition, schools should establish school-based counseling services for students, possibly by collaborating with volunteers from the Youth Union, the largest social-political organization of Vietnamese youth, at local universities. Teachers and parents should also participate in psychological education programs to raise awareness of how certain efforts with youth (i.e. pressure to perform, and harsh punishment), may be counterproductive. This may help to address some of the issues related to teacher and parent attitudes, and may allow for discussion of effective vs. abusive methods of discipline.

## Abbreviations

AUC: the area under the curve; CES-D: the Center for Epidemiology Studies Depression Scale; CI: Confidence interval; ESSA: the Educational Stress Scale for Adolescents; MCNV: the Medical Committee Netherlands-Vietnam; OR: Odds ratio; ROC: Receiver operating characteristic; SD: Standard deviation.

## Competing interests

The authors declare that they have no competing interests.

## Authors’ contributions

NTD, JB, JB, and CD jointly produced the idea and the study design. NTD, CD, PTT and PW developed the survey tools. NTD and PTT coordinated the surveys and data collection in the field. Data analysis was done by NTD, PTT, CD and PW. NTD was guided by CD and PW to produce the first draft and all authors contributed to the final version of the manuscript. All authors read and approved the final manuscript.

## Pre-publication history

The pre-publication history for this paper can be accessed here:

http://www.biomedcentral.com/1471-2458/13/1195/prepub
